# Perennial disaster patterns in Central Europe since 2000 and implications for hospital preparedness planning – a cross-sectional analysis

**DOI:** 10.1038/s41598-024-84223-4

**Published:** 2025-01-03

**Authors:** Maik von der Forst, Maximilian Dietrich, Felix C. F. Schmitt, Erik Popp, Markus Ries

**Affiliations:** 1 Heidelberg University, Medical Faculty Heidelberg, Department of Anesthesiology, Im Neuenheimer Feld 420, 69120 Heidelberg, Germany; 2 Heidelberg University, Medical Faculty Heidelberg, Center for Pediatrics and Adolescent Medicine, Pediatric Neurology and Metabolic Medicine, Im Neuenheimer Feld 430, 69120 Heidelberg, Germany

**Keywords:** Disaster, Mass casualty incident, Natural hazard, Hospital disaster planning, Critical infrastructure, Technological hazard, Public health, Epidemiology

## Abstract

The goal of this analysis is to describe seasonal disaster patterns in Central Europe in order to raise awareness and improve hospital disaster planning and resilience, particularly during peak events. Hospitals are essential pillars of a country’s critical infrastructure, vital for sustaining healthcare services and supporting public well-being—a key issue of national security. Disaster planning for hospitals is crucial to ensure their functionality under special circumstances. But the impact of climate change and seasonal variations in the utilization of hospital services are raising challenges. Therefore, the knowledge of perennial disaster patterns could help strengthen the resilience of hospitals. We conducted a cross-sectional analysis of the Emergency Events Database EM-DAT for disasters in Central Europe (Germany, France, Denmark, The Netherlands, Belgium, Luxembourg, Switzerland, Austria, Czech Republic, and Poland) between January 2000 and December 2023. Time distribution of disasters, patterns and longitudinal trends, were analyzed to discuss impact on disaster preparedness in hospitals. Out of 474 events, 83% were associated with a natural hazard and only 80 events (17%) were of technological cause. While technological disasters were spread equally over the whole year, the vast majority of disasters related to natural hazards (n = 394), i.e. storms (n = 178, 45%), floods (n = 101, 26%), and extreme temperatures (n = 93, 24%) peaked during summer and winter months. Fewer disasters were registered during autumn and especially spring seasons. More than 50% of the technological disasters were categorized in the transport accident subgroup. Technological disasters were spread equally over the whole year. Looking at the three most common disaster types, extreme temperatures, floods, and storms are clearly dominating and cause over 90% of the disasters due to natural hazards in central Europe. Overall, the number of events per year fluctuates without a clear trend, only the technological events appear to become less frequent with 70% (n = 56) of the registered disasters occurring in the first half of the study period (2000–2011). An overlap of hospital admissions due to seasonal effects and catastrophic events, mainly triggered by disasters of natural cause in vulnerable periods may lead to a partial collapse of the health care system. To close knowledge gaps, future comprehensive data collection is vital for informed decision-making. Awareness and preparedness are key: an "all-hazards" approach to manage diverse, potentially simultaneous seasonal threats is often the most versatile strategy for hospital emergency planning.

## Introduction

Hospitals are one of the main institutions of the health care system. As a part of the so-called critical infrastructure of a country, they are essential for the maintenance of public services, vital for sustaining healthcare services and supporting public well-being^1^. Hospital emergency planning is crucial to ensure that their functionality can be maintained, even under special circumstances. Strengthening the resilience of the critical infrastructure and including hospitals is one of the goals of the Sendai Framework for disaster risk reduction until 2030^2^. In different countries, hospitals are obligated to prepare plans for different potential emergencies such as mass casualty events, fires, or blackouts. There are existing national and international guidelines and reviews that define the most important scenarios to prepare for^3–7^. Most of the mentioned threats in these publications address isolated local problems (e.g. mass casualty, fire, …) and assume an intact infrastructure as well as the possibility of regional assistance through external authorities. However, a survey of 96 German hospitals showed that only about 25% of the hospitals have plans for floods or extreme weather events^8^. But in the last years in Europe, there were several events at a larger scale, like the “Ahrtal Flood” in the summer of 2021, different wildfires or heatwaves which compromised more than a single region at the same time and led at least partially to a destroyed or compromised infrastructure. Similar phenomena were also seen after the earthquake in Algeria in 2003 and the Indian Ocean tsunami of 2004, which led to a significant reduction of hospitals and other healthcare institutions^9^.

In addition to these disaster events partially due to climate change, the utilization of hospital services underlies seasonal differences. On the one hand, the summer months are associated with a higher number of heat-associated illnesses e.g. cardiovascular and renal diseases on the other hand during winter respiratory virus infections are common^10–12^. It is therefore essential to have an overview of the nature and the perennial pattern of disasters to prepare hospitals for these future challenges and help hospital administrators and emergency managers to prioritize resources. While there are many case reports and analyses for smaller events affecting hospitals (e.g. fire, traffic incidents, terroristic attacks, cybercrime), for Europe to our knowledge at the moment there is no analysis of which kind of events at the disaster level may predominantly affect hospitals^13–17^.

The goal of this analysis is to describe seasonal disaster patterns in Central Europe in order to raise awareness and improve hospital emergency planning and resilience, particularly during peak events. We therefore asked the following research questions:

1. In order to explore time-distribution of events: across the study period 2000–2023, were there particular months or seasons where disasters had particularly peaked, in the overall average?
2. In order to explore nature and pattern of events: which disasters were these?
3. In order to explore longitudinal trends: did the number of registered disasters change over time?
4. To raise awareness and to improve preparedness: what could be the impact of these recurring disaster patterns on hospitals and their emergency planning?

In summary, with the present cross-sectional study of the EM-DAT database, we aim to analyze and characterize disaster types in central Europe between 2000 to 2023 with a particular focus on perennial disaster patterns and outline the potential impact of these findings for future hospital emergency planning and better preparedness.

## Methods

A cross-sectional analysis of disaster events in the Emergency Events Database EM-DAT was conducted^18^. EM-DAT is a free open-access disaster database, that collects disasters from all over the world^19^. The study was conducted in 2024. Reported events between January 2000 and December 2023 (close of database 12 December 2023) in selected countries in Central Europe, which was defined as Germany and bordering countries (France, Denmark, The Netherlands, Belgium, Luxembourg, Switzerland, Austria, Czech Republic, and Poland) have been included. A regional approach was chosen due to various aspects. On the one hand, this increased the number of events to be considered, and seasonal effects become clearer. On the other hand, the consideration of individual countries has a greater range of fluctuation, which can be due to geographical conditions such as length of coasts, mass of mountains etc., but population size and area also play a role here. The selection of countries therefore was based on two particular assumptions: 1) there should be geographical proximity to make climate variables and exposure to the same kind of events comparable, and 2) the socio-economic status and technological development should be similar. Since the recording of disaster events in the EM-DAT register is contingent upon the availability of country reports, examining individual countries could also be subject to a significantly larger reporting bias, leading to distortion of the results. Events before 2000 were not taken into consideration because “*pre-2000 data is particularly subject to reporting biases*”, further the analyzed period of 23 years was considered the most current and best representative sample for the research questions^18^. The COVID-19 pandemic as an exceptional event affecting people worldwide was excluded from the analysis, because it has special emergency implications due to its magnitude and is considered to be different from the usual underlying disaster pattern.

All listed disaster events were defined as situations that: *“overwhelm local capacity, necessitating a request to the national or international level for external assistance; an unforeseen and often sudden event that causes great damage, destruction, and human suffering.”*^18^*.* To be listed an event has to further meet at least one of the following EM-DAT Inclusion Criteria: at least ten deaths (including dead and missing), at least 100 affected (people affected, injured, or homeless), a call for international assistance or an emergency declaration^18^. Primary data sources for EM-DAT include “UN agencies, non-governmental organizations, reinsurance companies, research institutes, and press agencies”^18^.

Disasters are listed with four levels of depth, so events are divided into groups, subgroups, types, and subtypes. The three EM-DAT disaster groups are ‘Natural’, ‘Technological’, and ‘Complex’. The IRDR Peril Classification and Hazard Glossary informs EM-DAT’s classification of natural hazards, organizing them into six main subgroups: Geophysical, Hydrological, Meteorological, Climatological, Biological, and Extra-terrestrial^20^.

Data were downloaded from the EM-DAT website on 12^th^ December 2023 and considered events including November 2023 and then exported into an electronic database system (Microsoft Excel®, Microsoft Deutschland GmbH, Unterschleißheim, Germany). Variables for analysis included: [Disaster Group, Disaster Subgroup, Disaster Type, Country, Start Year, Start Month, Start Day, End Year, End Month, End Day, and Total Deaths]. The statistical analysis and figures were performed with SPSS (Statistical Product and Services Solutions, Version 25, SPSS Inc., Chicago, IL, USA) and GraphPad Prism (Version X, GraphPad Software, La Jolla, USA). Standard methods of descriptive statistics were applied in the analysis of the complete dataset. Further we compared disaster events per month with each other calculating relative risks with the corresponding confidence intervals and determining significance using contingency analysis and Fisher’s exact test for statistical significance. A p-value of < 0.05 was considered statistically significant.

We compared, on the one hand, the ‘disaster groups’ natural and technological, and on the other hand ‘disaster types’ according to the EM-DAT nomenclature, to gain a clearer understanding of the distribution and characteristics of different disaster events The perennial pattern of disasters was elaborated by attributing all disasters across the overall analysis period to their month of occurrence, irrespective of the year. Further, the disasters were divided into types following the EM-DAT nomenclature, and the perennial pattern of types was analyzed in the same matter. Every disaster event was listed by the country that was affected and then compared to the ground area and the number of inhabitants of every country.

Also, the number of events per year as well as the duration in months of every event were analyzed. Further the total number of events for every single year was analyzed throughout the whole study period.

For a comparison of disaster numbers and the total Inhabitants of the different countries the German Website statista.de was used^21^. The study fulfills the requirements of the STROBE statement^22^. Ethics: As a descriptive analysis based on pre-existing data without direct human or animal research involvement, the present study did not require an Institutional Review Board Ethics Committee review.

## Results

Since 1^st^ January 2000, 474 events were registered in EM-DAT. More than half of all analyzed disasters (54%) occurred from 2000–2011 during the first half of the study period. Overall, the number of disasters in central Europe, as defined for the underlying study, seems to decrease over time.

### Seasonal distribution of disasters (2000–2023)

**Fig. 1:**
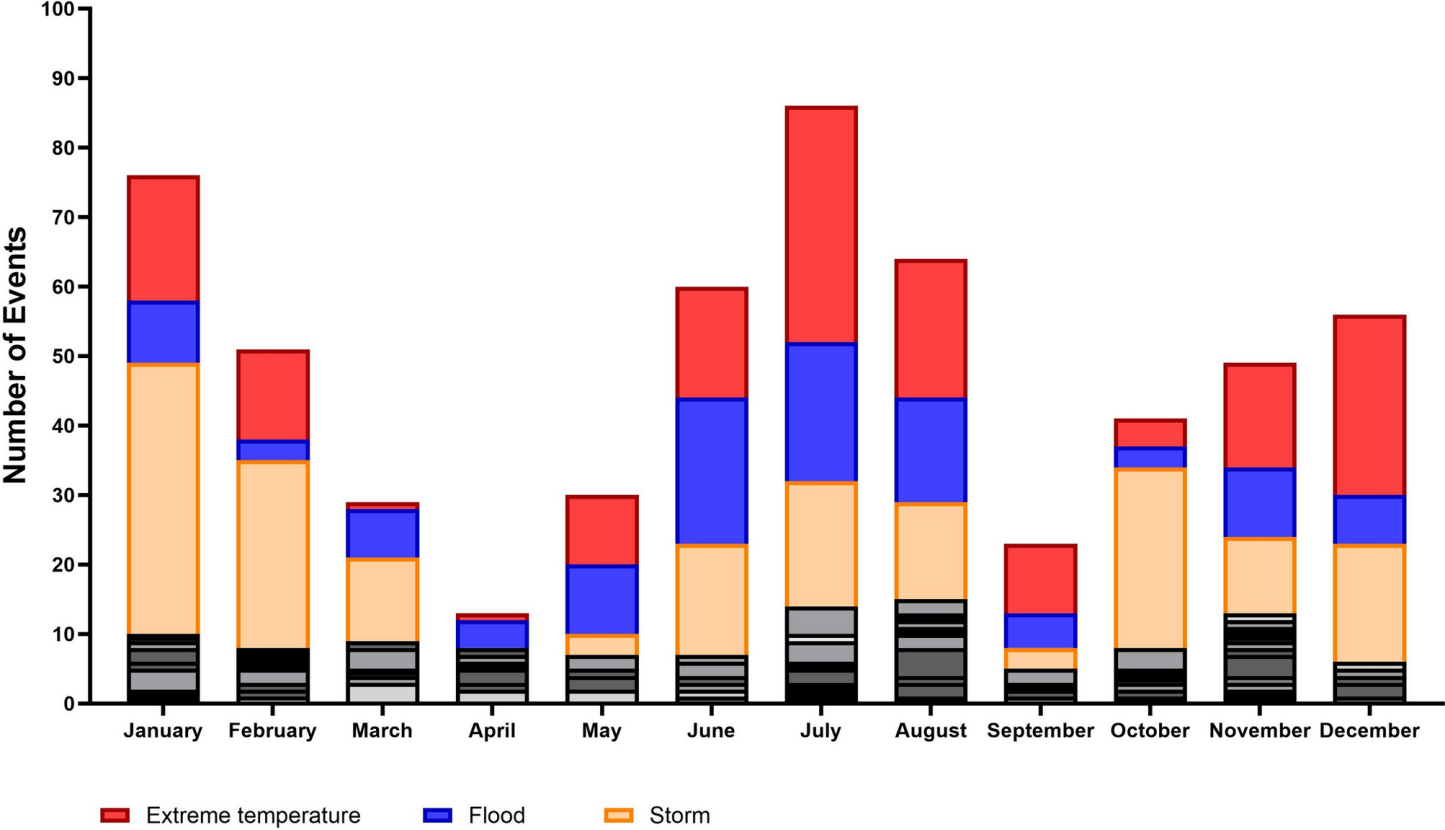
Perennial Pattern of Disasters in Central Europe by Month of Occurrence between January 2000 and November 2023. The figure shows the monthly distribution of disaster events in the whole research period.

The number of events categorized by month of occurrence across the overall analysis period shows that the disaster peaks are situated during summer and winter months, while fewer disasters are registered during autumn and especially spring seasons. Compared to technological events the frequency of disasters related to natural phenomena was much higher, since 2000 there were registered 394 events in EM-DAT. The analysis of natural hazard disasters showed a clear seasonal pattern in their occurrence (Fig. 1).

### Nature and types of disasters

**Fig. 2:**
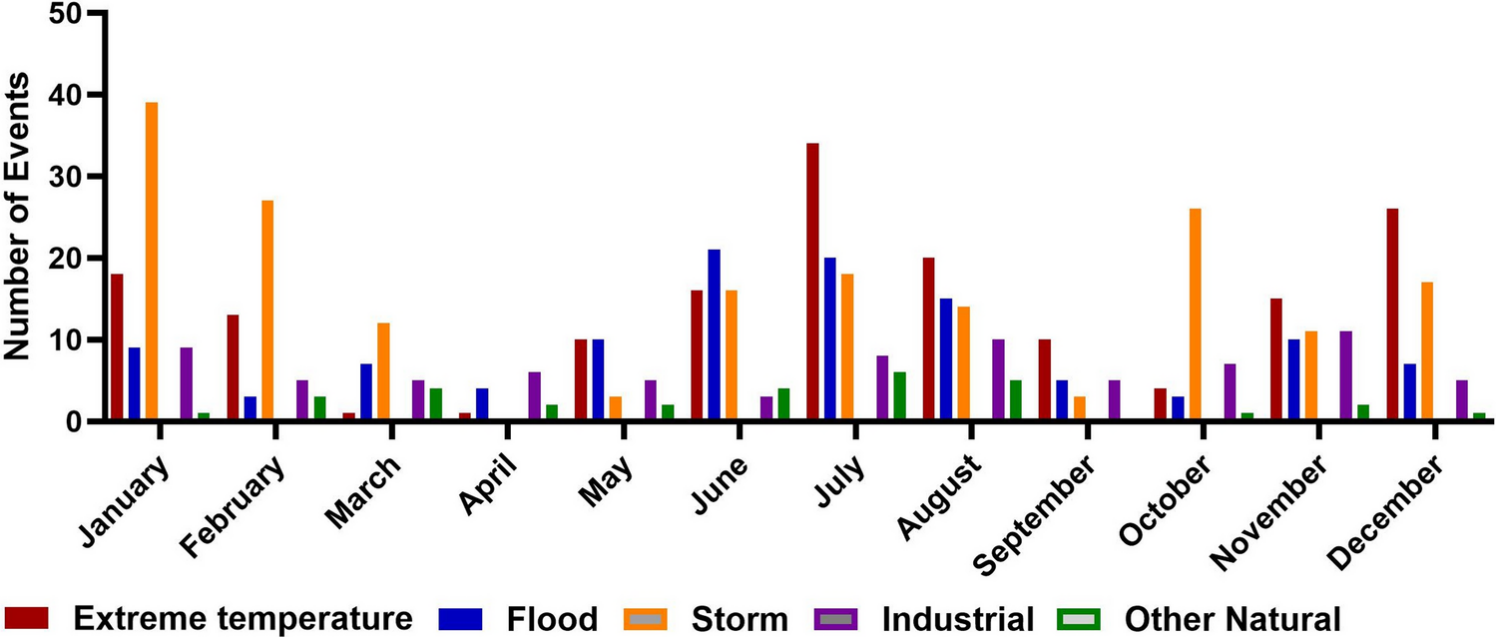
Overall Number of Disasters in Central Europe between 2000 and 2023, categorized by Month of the Year. The graph shows the number of events per month with a single column for every disaster type. While the three disaster types extreme temperatures (= red), floods (= blue) and storms (= yellow) are shown separately, for a better visibility all other disaster types are only divided into “technological” (= magenta) and “other natural” (= green).

Going more into detail reveals that these periodical fluctuations can be associated predominantly with the three main types of natural disasters, i.e., extreme temperatures, flood, and storms (Fig. 2). The months March, April, and May show the lowest incidence of the main disaster types in central Europe, further also September, October, and November from 2000–2023 were less affected by disasters than winter or summer months (Fig. 2). Especially it is to mention that winter and summer are also the seasons in which events of different types tend to occur simultaneously.

While there were some minor causes like drought (n = 2), earthquake (n = 3), epidemic (n = 4), mass movement (n = 8), and wildfire (n = 5), which taken together represent only 5.6% of all listed natural disasters, the vast majority of disasters were due to storm (n = 178, 45%), flood (n = 101, 26%) and extreme temperatures (n = 93, 24%).It appears that while storms are most frequent in winter, more floods are registered during the summer months (Fig. 2). For extreme temperatures there are two peaks, i.e., extreme heat in the summer and one extreme cold in the winter months (Fig. 2). Importantly, while the absolute number of cold events is higher (n = 52 vs. 41) than extreme heat, the adverse impact regarding death is nearly completely associated to heat events (97% of all extreme temperature deaths). Considering all events, there were 60 that had a duration > 1 month from start to end, 55 of these belong to the three dominating disaster types storm, flood, and extreme temperature. The maximum duration of a disaster was 5 months (extreme temperature). In general, extreme temperature disasters last longer than floods and storms. Some major characteristics of the three main disaster types are shown in Table 1.

**Table 1:** Main season and deaths associated with disasters due to extreme temperature, flood, and storm in Central Europe between 2000 and 2023. Relative Risk and Fisher’s exact test were calculated for the incidence of a disaster in the two months with most events compared to those two with the fewest events.

	Extreme temperature	Flood	Storm
N =	93 (41 × Heat/52 × Cold)	101	178
Main Season (% of total Events)	Winter 37% Summer 33%	Summer 50%	Winter 47%
Death (n)	64,456 (Heat 97%)	573	470
Events/Month Two Max vs Two Min	July/August vs March/April	June/July vs September/October	January/February vs April/May
Relative Risk (CI 95%)	7.74 (2.28-28.39)	2.25 (1.16–4.54)	7.45 (2.75–21.87)
Fisher’s exact test	p < 0.0001	p < 0.05	p < 0.0001

### Comparison of disasters caused by natural or technological hazards

In total most of the analyzed disasters were associated with a natural cause and only 80 events (= 17%) were technological. More than 50% of the technological disasters were categorized in the transport accident subgroup (aircraft n = 9, rail n = 13, road n = 19, water n = 2). The remaining events (n = 37) were all the subgroup industrial and miscellaneous accidents. In detail, there were mostly fires (n = 14) explosions (n = 13), or collapses (n = 4). There were no substantial differences between the analyzed countries, whereas, of interest, the bigger industrialized ones in total had more technological events (e.g. France n = 23, Germany n = 19). Looking at the different months, there was a slight fluctuation in technological disasters over the whole year. The months January, July, and November have more events in total, but these could not be attributed to specific causes and seems coincidental (Fig. 2).

### Differences of disasters between the analyzed countries

As expected, the number of disasters was different between the selected countries. France (n = 137) and Germany (n = 83) showed the highest total number of disasters. Comparing these data with the area and the population size of the countries it results that both France (2.1 events/M inhabitants) and Germany (0.96 events/M inhabitants) are together with Denmark (1.7 events/M inhabitants) the three countries with a relatively smaller number of disasters, while e.g. in Luxembourg there were 9.2 events/M inhabitants^21^.

**Fig. 3:**
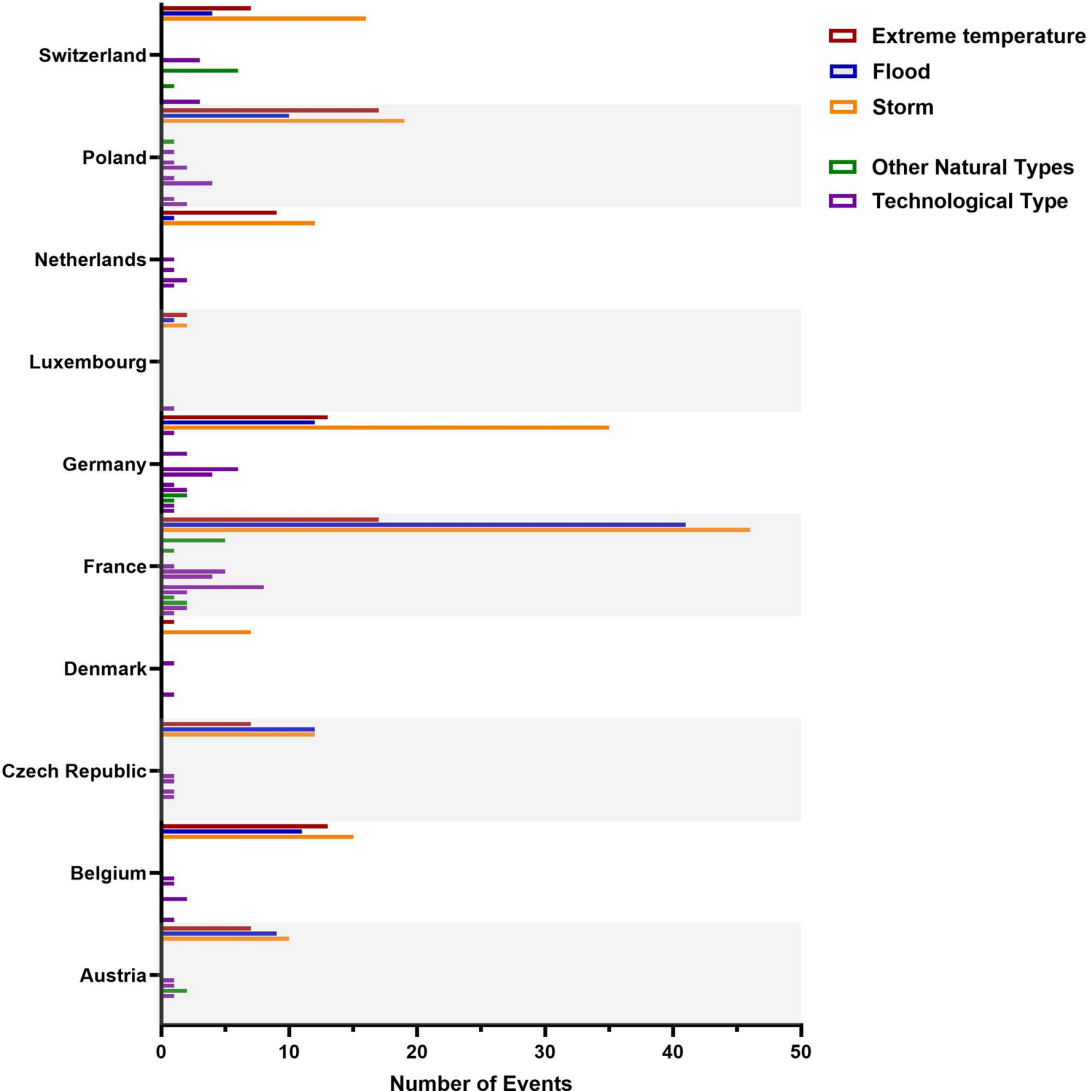
Absolute number of disasters between 2020 and 2023 categorized by country and disaster type. The graph shows the number of events per country with a single column for every disaster type. While the three disaster types extreme temperatures (= red), floods (= blue) and storms (= yellow) are shown separately, for a better visibility all other disaster types are only divided into “technological” (= magenta) and “other natural” (= green).

The three main types of disasters—extreme temperatures, storms, and floods—were the most common across nearly every country analyzed. There are only a few exceptions regarding flood events. Denmark (0% of all events), the Netherlands (4% of all events), and Switzerland (10% of all events) had relatively few flood disasters registered in the database (Fig. 3).

### Longitudinal trends in disaster frequency

**Fig. 4:**
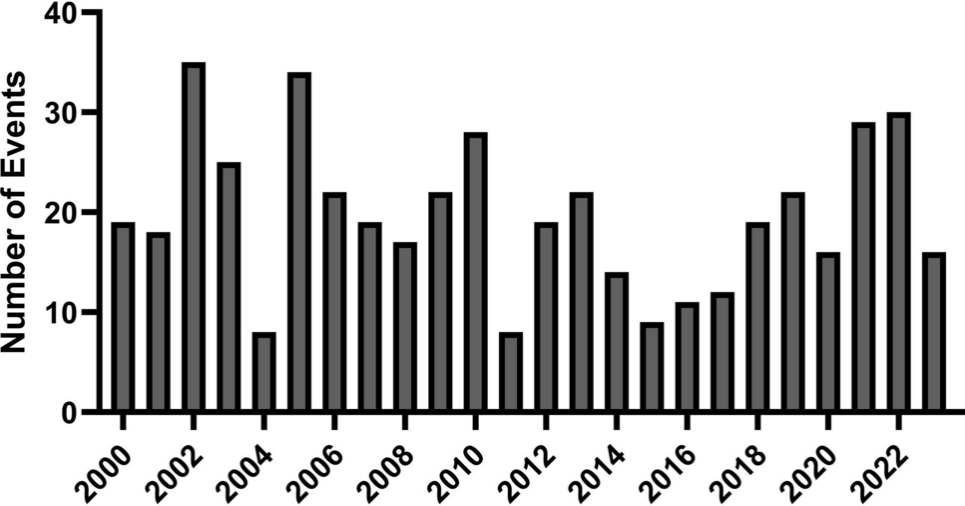
Overall Number of Disasters 2000 – 2023 in Central Europe categorized by Year of Observation.

Looking at the distribution of the number of registered disasters per year (Fig. 4) in the countries observed, there is no clear trend. There are strong annual fluctuations, which is why a longer observation period is always required for generalizable statements. Dividing the analyzed period into equal parts (2000–2011 & 2012–2023) it appears that technological events are becoming less frequent and 70% (n = 56) of the registered disasters happened between 2000 and 2011.

## Discussion

The present analysis points out the perennial pattern of disasters across central Europe between 2000 and 2023 and highlights critical peak seasons necessitating hospital preparedness. Notably, our examination reveals that the summer and winter months emerge as the most vulnerable periods, characterized by a prevalence of extreme temperatures, floods, and storms. Particularly alarming is the disproportionate loss of life attributed to heatwaves during the summer months. While other regions of the globe have grappled with the impact of natural hazards such as floods, hurricanes, and droughts on healthcare infrastructure for decades, Central Europe has, fortunately, been largely spared by such events in recent years^23–27^. Preparation and precaution are nevertheless essential to diminish the number of deaths in these potentially upcoming disasters^28^. An essential factor is that public healthcare could be affected much longer than the event itself^29^. Different events of the last few years e.g. the “Ahrtal Flood” during summer 2021 confirmed this perception, some hospitals that were hit by the flood and in part had to be evacuated, were not able to get back to their usual business for several months^30–32^. Unfortunately, the level of hospital preparedness varies, and probably due to the lack of legislation or financial issues, it is often insufficient^33^. Especially in industrialized countries compared to developing countries the focus of disaster planning lies less on natural hazards^34^.

The analysis of registered disasters in central Europe during the last 23 years showed that the main group is due to natural hazards (Fig. 2) which affect the different countries similarly (Fig. 3). The results of the ongoing analysis further indicate that especially during summer and winter months (Fig. 1), the incidence of disaster events is higher than in spring and autumn. Both are seasons where the demand for health services usually is already high and hospitals are often working at their limits e.g. due to heat or the annual epidemics of different respiratory viral diseases^10^. Looking at the three most common disaster types, extreme temperatures, floods, and storms are clearly dominating and cause over 90% of the disasters related to natural hazards in central Europe (Fig. 2). In the hospital disaster planning literature less than 10% and in a systematic review by Hasan et al. only two of thirteen articles were dealing exclusively with these three hazards^34,35^.

Taken together extreme temperatures appear during the summer and winter, whereas especially heat periods are a major health problem and cause a significant number of deaths^36–41^. This is mostly due to heat-related illness including e.g. cardiovascular and respiratory complications as well as renal failure and negative impact on fetal health^11,42^. The distribution analysis reveals that floods predominantly occur during the summer months, while storms are more frequent in late autumn and winter. This pattern suggests the possibility of concurrent disaster types (e.g., a heat wave coinciding with a flood) (Fig. 1). Due to climate change, these most frequent hazards are likely to increase in frequency and impact a broader population across the evaluated region in the future^25^. An overlap of hospital admissions due to seasonal effects and disaster events may lead to a partial collapse of the healthcare system.

As part of the critical infrastructure, it makes sense for hospitals to have plans in place for exceptional events. In some countries, these disaster plans are also required by law. The content and design can vary; in principle, a distinction must be made between the “all-hazards” approach and specific plans for each event. The special skills required, e.g. increasing bed capacity, evacuation, setting up management structures, etc., also depend on the type of event. In total compared to other topics there is still only a few scientific literature regarding the relationship of disaster events and hospital disaster planning. Also, if there is a rising number of scientific articles coming up especially after larger disaster events, the topic as a whole still seems to be neglected^43^. Different reviews indicate that in the majority of hospitals, some crucial aspects of disaster planning are missing, these include especially dead body handling, waste management, transport, and access routes, which are relevant mostly in disaster situations caused by natural hazards leading also to a destroyed infrastructure^34^. A systematic review by Hasan et al. had similar findings, screening over 2500 publications led to 13 peer-reviewed articles focusing on hospital surge capacity preparedness. Overall, missing “stuff” and staff, the lack of standardized assessment tools, and system related barriers were identified as the limiting factors to preparedness for surge capacity^35^. An analysis from Melnychuk et al. found 363 peer-reviewed publications including the grey literature. Sorted by type it shows that there are 13 different kinds of threats and over > 45% of the peer-reviewed literature belong to the category “Others”, with the conclusion that hospitals are often confronted with similar vulnerabilities, threats and security gaps during disasters^44^.

Looking at the results of the present study and the findings of the literature it should be of high interest and priority to prepare hospitals for the three main disaster types extreme temperature, flood and storm as well as potentially overlapping disasters (e.g. associated pandemic or mass casualty events)^45^. However, it remains unclear why intensive preparation has not taken place for a long time, because from a financial perspective, there can actually be no rational arguments preventing preparation for these events^46^. Regarding that in the U.S. weather and climate disasters since 1980 caused total damage that exceeds $2.655 trillion, every money spent on prevention is well invested, because disaster prevention is cheaper than disaster response and recovery^47^.

The “all-hazards” approach mentioned above is already being applied in many parts of the world. As early as the early 1990s, Quarantelli et al. questioned the cause-specific approach to disasters and criticized among others the division into natural and technical disasters. A more generalist approach was propagated, which focuses more on the social and institutional consequences of events^48–50^. As in any other VUCA (“Volatile, Uncertain, Complex, and Ambiguous”) situations, hospitals should be prepared to adapt quickly, leverage established networks, secure critical resources, and make rapid decisions to effectively manage diverse disaster scenarios^51^. Specifically, to deal with consequences of different and potentially simultaneous hazards using an “all-hazards” approach could be promising and has often been seen as the most versatile and flexible strategy for hospital disaster planning^52,53^. Preparing for “all-hazards” means that the processes of disaster planning are organized without knowledge of the specific event. On the one hand, this model has some advantages, especially in case of disasters that previously were not considered in the plans of a single hospital, but on the other hand it could also have potential negative effects regarding the preparation of hazard-specific tasks^54^. A mixture of both strategies e.g. by creating a toolbox including hazard-specific protocols but using an “all-hazards” approach as a backbone for command and control, communication, and alert issues could strengthen resilience of hospitals. For hospitals to operationalize disaster preparedness, Verheul et al. developed a nonagon with essential components^55^. Further it could be helpful to consider the 2015 updated Hospital Safety Index tool, developed by the World Health Organization and the Pan American Health Organization, which is the most used tool for hospital disaster preparedness assessment^56^**.**

Considering the numerous deficits mentioned and the complexity of the topic, hospitals should engage experts who develop a hospital disaster plan/ business continuity plan (often used synonymously)^57^. Global learning could generate synergies: in other parts of the world, the high frequency of climate-associated disaster events and the analysis of their potential damages as well as community disruptions already led to the development of toolkits to prepare the healthcare system for changing climate^58–60^.

## Limitations and future directions

This analysis relies on the accuracy of the data collection in EM-DAT. The conceptual focus of EM-DAT comes with limitations when addressing complex and compound events and situations. The data collection in EM-DAT considers pre-existing data from a variety of external sources with unchangeable elements which limits the possibility of a more specific analysis. It cannot be excluded that the recording of disaster events may be incomplete^61^. EM-Dat describes three main data quality issues: missing events, events existing with missing values, events with inaccurate attributes. Accordingly, excluding pre-2000 data from trend analyses based on EM-DAT is recommended. The completeness of EM-DAT reflects the extent of coverage by specific sources. However, gaps and quality concerns persist. Further there are possible accounting biases are for example that, insured damages are typically reported more often than uninsured damages, leading to a geographic ascertainment bias in areas with limited insurance coverage, such as Africa. Nevertheless, during the analysis period covered in this report, data acquisition into EM-DAT was performed prospectively.

The number of deaths, although being considered as a hard endpoint in clinical research, can at best be a surrogate parameter for the impact on hospitals during a disaster. Nevertheless, this parameter has been included in the analysis to illustrate the varying impact of individual disasters on health. In particular, the large number of heat-related deaths is often associated with pre-existing morbidity and significantly distinguishes this type of disaster from the others. Furthermore, EM-DAT does not include any specific parameters related to hospitals, so surrogate parameters must be used for estimation purposes.

The number of injured is also recorded in the register; however, after careful review, this variable appears to be incomplete. Therefore, in order to avoid potentially confusing reporting bias driven issues, we have decided not to show this parameter. It should be noted that EM-DAT Database does not specify the temperatures in degrees for the definition of the parameter “extreme temperature”. The duration of heat or cold waves is defined as two or more days. Another relevant limitation is that some disasters which affected more than one country at the same time were listed as a single entry in EM-DAT for every country and the given data did not offer the possibility to group these events reliably. Therefore, the same disaster appears once for every single affected country, which may lead to an overestimation. On the other hand, the advantage of this method is that events hitting larger geographical areas appear with a higher impact by their higher single number. Future research could further compare the distribution of disasters in the studied areas with other countries or continents to identify broader patterns and differences. In addition, pediatric aspects will be of importance^62^.

Taken together for the future of disaster databases, it could be important to place an additional focus on hospital disaster planning and to introduce corresponding items. In Germany, a non-profit project to register events that activate hospital disaster plans was launched a few months ago for precisely this purpose (dakep.krisenregister.de). Further it is very important to have a disaster information registration system in each country, to mitigate reporting biases.

## Conclusion

In conclusion, this research highlights several critical insights:

First, throughout the year, there is a relatively steady number of technological disasters, overlaid by seasonal peaks of natural hazards, with increased heatwaves, storms, and floods in summer and cold spells and storms in winter.

Second, an overlap of hospital admissions due to seasonal effects and catastrophic events, mainly triggered by natural disasters in vulnerable periods, so called „twin threats “ may lead to a partial collapse of the health care system.

Third, awareness and preparedness are key: using an "all-hazards" approach to manage diverse, potentially simultaneous seasonal threats is often the most versatile strategy for hospital emergency planning.

Fourth, there is a lack of solid transparent epidemiological foundations for hospital disaster planning and EM-DAT, one of the largest databases in this area, lacks crucial items (e.g. hospitalized patients, outpatient consultations, transport by the emergency services, destroyed health facilities, age stratified data including pediatric aspects) in order to assess impacts on the health sector. In the future, it will be essential to record these data comprehesively across all countries to enable better-informed decision making.

For practitioners and researchers, these findings underscore the need for improved awareness and preparedness strategies, enhanced data collection, and a collaborative, global approach to address the evolving challenges in hospital disaster planning.

## Data Availability

The datasets generated during and analysed during the current study are available in the EM-Dat repository. EM-DAT contains data on the occurrence and impacts of over 26,000 mass disasters worldwide from 1900 to the present day. The Centre for Research on the Epidemiology of Disasters (CRED) distributes the data in open access for non-commercial use. https://public.emdat.be/
